# Cross-species high-resolution transcriptome profiling suggests biomarkers and therapeutic targets for ulcerative colitis

**DOI:** 10.3389/fmolb.2022.1081176

**Published:** 2023-01-05

**Authors:** Reza Yarani, Oana Palasca, Nadezhda T. Doncheva, Christian Anthon, Bartosz Pilecki, Cecilie A. S. Svane, Aashiq H. Mirza, Thomas Litman, Uffe Holmskov, Claus H. Bang-Berthelsen, Mogens Vilien, Lars J. Jensen, Jan Gorodkin, Flemming Pociot

**Affiliations:** ^1^ Translational Type 1 Diabetes Research, Department of Clinical Research, Steno Diabetes Center Copenhagen, Gentofte, Denmark; ^2^ Novo Nordisk Foundation Center for Protein Research, University of Copenhagen, Copenhagen, Denmark; ^3^ Center for non-coding RNA in Technology and Health, University of Copenhagen, Copenhagen, Denmark; ^4^ Department of Veterinary and Animal Sciences, University of Copenhagen, Copenhagen, Denmark; ^5^ Department of Cancer and Inflammation Research, Institute of Molecular Medicine, Faculty of Health Sciences, University of Southern Denmark, Odense, Denmark; ^6^ Department of Pharmacology, Weill Cornell Medicine, Cornell University, New York, NY, United States; ^7^ Department of Immunology and Microbiology, University of Copenhagen, Copenhagen, Denmark; ^8^ Research Group for Microbial Biotechnology and Biorefining, National Food Institute, Technical University of Denmark, Kgs. Lyngby, Denmark; ^9^ Department of Gastroenterology, North Zealand Hillerød Hospital, Hillerød, Denmark; ^10^ Department of Surgery, North Zealand Hospital, Hillerød, Denmark; ^11^ Copenhagen Diabetes Research Center, Department of Pediatrics, Herlev University Hospital, Herlev, Denmark; ^12^ Department of Clinical Medicine, Faculty of Health and Medical Sciences, University of Copenhagen, Copenhagen, Denmark

**Keywords:** ulcerative colitis, coding RNAs, non-coding RNAs, conserved expression signature, biomarkers

## Abstract

**Background:** Ulcerative colitis (UC) is a disorder with unknown etiology, and animal models play an essential role in studying its molecular pathophysiology. Here, we aim to identify common conserved pathological UC-related gene expression signatures between humans and mice that can be used as treatment targets and/or biomarker candidates.

**Methods:** To identify differentially regulated protein-coding genes and non-coding RNAs, we sequenced total RNA from the colon and blood of the most widely used dextran sodium sulfate Ulcerative colitis mouse. By combining this with public human Ulcerative colitis data, we investigated conserved gene expression signatures and pathways/biological processes through which these genes may contribute to disease development/progression.

**Results:** Cross-species integration of human and mouse Ulcerative colitis data resulted in the identification of 1442 genes that were significantly differentially regulated in the same direction in the colon and 157 in blood. Of these, 51 genes showed consistent differential regulation in the colon and blood. Less known genes with importance in disease pathogenesis, including *SPI1, FPR2, TYROBP, CKAP4, MCEMP1, ADGRG3, SLC11A1,* and *SELPLG,* were identified through network centrality ranking and validated in independent human and mouse cohorts.

**Conclusion:** The identified Ulcerative colitis conserved transcriptional signatures aid in the disease phenotyping and future treatment decisions, drug discovery, and clinical trial design.

## Introduction

Ulcerative colitis (UC) is an inflammatory bowel disorder mainly affecting the large intestine. Most often, UC initiates from the rectum, affects the mucosal lining, and is limited to the innermost layers of the large intestine. Multiple pathogenic factors, including numerous susceptibility gene variants, environmental factors, changes in the gut microbiota, and a dysregulated immune response, are all associated with UC. Despite this recognition and the identification of apparently relevant factors, a complete understanding of UC pathogenesis still needs to be reached, and thus, treatment may not be optimal. An important reason for this unsatisfactory situation is the currently limited comprehension of the genuinely relevant components of UC immuno-pathogenesis. Thus, given the complex nature of UC, the study of animal models is crucial. A big challenge in using animal models for UC is whether the model’s genes and molecular pathways are analogous to humans. Although many coding and non-coding genes between humans and mice are highly similar (Hong et al., 2017), they might be regulated differently, and the pathophysiology may differ. The expression of both differentially regulated coding and non-coding genes contributes to disease pathology (Casamassimi et al., 2017; Lekka and Hall, 2018). Differentially regulated long non-coding RNAs (lncRNAs) and microRNAs (miRNAs) are linked to the change in the expression of many protein-coding genes (PCGs) and, subsequently, the development of disease. Many studies are made without taking the non-coding RNAs, i.e., disease-related pathways and differentially expressed genes, into account, and thereby, fundamental and essential players in the disease regulatory networks may be overlooked (Hasler et al., 2017).

Preclinical murine models have been used for *in vivo* assessments of UC. One of the best-established UC models is the dextran sulfate sodium (DSS) model (Chassaing et al., 2014; Eichele and Kharbanda, 2017). DSS is a negatively charged polysaccharide with colitogenic properties that induce colitis when given orally (Chassaing et al., 2014). Although the mechanisms by which DSS causes intestinal inflammation are not fully understood (Okayasu et al., 1990), it seems that the exposed colonic monolayer of epithelial cells in the large intestine, specifically the distal colon, are being progressively eroded, and the lamina propria barrier integrity is compromised, in which an enormous number of microorganisms live. Therefore, inflammation is triggered following bacteria (and their products) translocation across the damaged intestinal wall. However, to our knowledge, a comprehensive investigation of the DSS model’s coding and non-coding colonic and blood transcriptome and its comparison with human UC has not been performed as of today.

We, therefore, set out to characterize the transcriptomic landscape of the DSS-UC mouse model by performing transcriptomic profiling of PCGs, lncRNAs, and miRNAs from colon and whole blood using deep RNA sequencing. We identified differentially expressed lncRNAs and underscored their functional importance by annotating their expressed neighboring PCGs. Then we obtained the targets for the differentially regulated miRNAs and identified the biological processes that these targets are affecting. Further, we assessed the similarity between the experimental model and human UC. To this aim, several high-quality publicly available human UC colonic and blood transcriptomic studies, including our previous study, were selected, data obtained, processed, and cross-species UC transcriptomic landscape was compared. Our results highlight widespread differential regulation of coding and non-coding genes in the mouse and human UC models with marked similarities in both tissues, specifically in the colon. We also explored the underlying molecular pathways and networks through which these genes may contribute to disease development and progression. This study offers an excellent opportunity to investigate the role of novel and previously identified differentially regulated genes in UC. Moreover, cross-species high-resolution UC transcriptome comparison suggests new biomarkers and therapeutic targets and improves molecular phenotyping.

## Methods

### Mouse model, RNA sequencing, data processing, and differential expression analysis

Ulcerative colitis was induced using dextran sulfate sodium salt (DSS) in C57BL/6 male mice. The control group was time-matched and received the same drinking water without DSS. The disease activity index (DAI) was recorded. RNA was extracted from the colon and whole blood, and total and small RNA were sequenced using the Illumina HiSeq 4000 system. Gene-level quantification corresponding to the total RNA-Seq data was obtained using Stringtie (Pertea et al., 2016), and mature miRNA expression was quantified using miRDeep2 (Friedlander et al., 2012). Differential expression was performed using DESeq2 (Love et al., 2014) (Supplementary Methods). The raw total and small RNA-Seq data have been deposited in the Gene Expression Omnibus (GEO) database with accession number GSE155303.

### Functional enrichment and network analysis of differentially expressed genes

Functional enrichment analysis on the significantly differentially expressed (SDE) genes (FC > 2, padj ≤ 0.05) was performed using stringApp (Doncheva et al., 2019) in Cytoscape (Shannon et al., 2003) as well as Ingenuity pathway analysis (IPA) software (Krämer et al., 2014).

To suggest a functional role for SDE lncRNAs, we performed enrichment on their genomic neighbor genes situated within a span of 100 kb upstream and downstream of the lncRNA. For SDE miRNAs (padj ≤ 0.05), we retrieved a set of miRNA-target genes and performed enrichment on them (Supplementary Methods).

StringApp was used to retrieve STRING networks (Szklarczyk et al., 2019) in Cytoscape for the set of SDE PCGs in mouse colon and blood and for the SDE PCGs conserved in humans. Furthermore, weighted centrality analysis was performed on their STRING network to rank the SDE genes common between mouse and human colon and blood based on their importance (Supplementary Methods).

### Comparison between mouse and human data

To compare our mouse data with data from UC patients, we obtained and processed data from eight publicly available studies (Van der Goten et al., 2014; Mirza et al., 2015; Vanhove et al., 2015; Lin et al., 2016; Mo et al., 2018; Haberman et al., 2019; Ostrowski et al., 2019). A set of SDE genes in the colon and blood was obtained by combining the different datasets, requiring each gene to be consistently SDE (padj ≤ 0.05) in at least two datasets. For the small RNA-Seq, we only used two datasets and required that each miRNA is SDE (padj ≤ 0.05) in at least one of these datasets. The final SDE human lists were intersected with the mouse SDE blood and colon gene sets (padj ≤ 0.05) using orthology relationships extracted from Ensembl (release 97) (Yates et al., 2020) and HCOP database (Wright et al., 2005; Seal et al., 2011) for lncRNAs (Supplementary Methods).

### Validation of SDE genes by quantitative real-time PCR

The expression of 19 selected SDE genes common in mouse and human colon and blood was validated by quantitative real-time PCR (qRT-PCR). Colon and blood cDNAs from 5 DSS-UC mice, five control mice, 10 UC patients, and six controls were used for validation. Detailed information on patient demographics and primer sequences used for qRT-PCR can be found in Supplementary File S1, and procedure detail in the supplementary section. The expression of each gene tested was represented as a FC using the 2^−ΔΔCT^ or 2 (-Delta Delta C(T)) method (Livak and Schmittgen, 2001). *GUSB*, *B2M*, *ACTB,* and *TBP* were the reference genes for normalization.

## Results

### Transcriptional profile of UC mouse model

Successful colitis induction in mice was confirmed by combined DAI score, measuring inflammatory markers, histological and micro positron emission tomography imaging analysis (Supplementary Figure S1). For the total RNA-Seq, 40–66M paired-end reads were obtained per sample, with 39–58M in the colon and 17–33M in blood, uniquely mapping to the mouse genome. For the small RNA-Seq after cleaning, 15–25M reads were obtained, where 14–20M reads mapped to the set of mouse miRNAs annotated in miRBase v.22. Principal component analysis (Supplementary Figure S2) shows a clear separation of the disease and control groups on the first principal component in total RNA-Seq colon and blood, as well as small RNA-Seq colon. The differential gene expression pattern strongly supports the separation between the groups, suggesting that global gene expression differences can be detected between UC and healthy controls (Table 1 and Supplementary File S2).

**TABLE 1:** Numbers of SDE genes identified in colon and blood of DSS-UC mice versus control. (For PCGs and lncRNAs |log2FC| > 1, padj ≤ 0.05 and for miRNAs FC > 1.5, padj ≤ 0.05).

	Colon	Blood
PCGs	2479 (▲1897, ▼582)	518 (▲500, ▼18)
lncRNAs	282 (▲135, ▼147)	44 (▲40, ▼4)
miRNAs	73 (▲39, ▼34)	10 (▲3, ▼7)
*Others	300 (▲201, ▼99)	54 (▲52, ▼2)

*e.g. Pseudogenes, TEC, snoRNA, miscRNA, etc. Upregulation ▲, Downregulation ▼.

### UC mouse colon and blood transcriptomics signature

**FIGURE 1:**
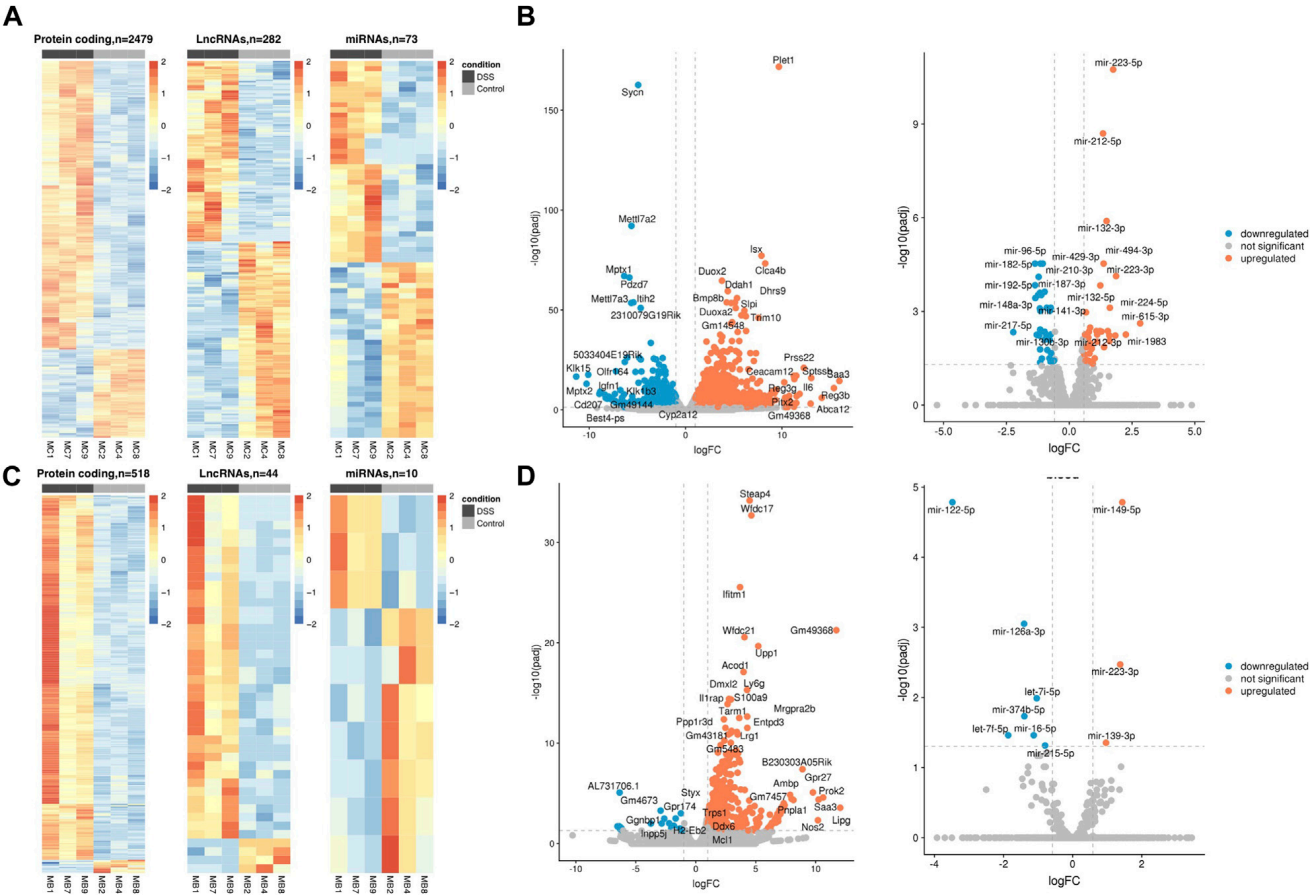
DSS-UC mouse transcriptional signature. Heatmaps of SDE PCGs, lncRNAs, and miRNAs, based on z-scores of normalized log counts for **(A)** colon and **(C)** blood. Volcano plots log2FC ratio on the x-axis versus -log10 padj on the y axis for colon **(B)** and blood **(D)**. (UC, *n* = 3 and CO, *n* = 3).

We identified 3,061 and 623 SDE genes from the total RNA-Seq and 73 and 10 SDE miRNAs from the small RNA-Seq data comparing UC mice colon and blood with control, respectively (Table 1). While SDE PCGs are mainly upregulated in both tissues, lncRNAs in the colon are equally up- and downregulated, and the miRNAs in the blood are mostly downregulated. The RNA expression profile of the SDE genes and miRNAs, obtained by hierarchical clustering, is shown in heatmaps for the colon and blood (Figures 1A, C). In addition, the SDE genes and miRNAs are highlighted in the volcano plots for the colon and blood (Figures 1B, D).

From the SDE genes in the colon, we detected 116 genes with a human ortholog situated within 100 kb distance of the known Informatory bowel disease (IBD)-risk loci (Jostins et al., 2012; de Lange et al., 2017), i.e., 110 PCGs, two lncRNAs, and four other RNAs. In blood, 30 SDE genes, all PCGs, were IBD-risk loci associated (Supplementary File S3). In addition, several of the SDE genes, including *Reg3b, Abca12, Sptssb, Prss22, Pitx2, Ceacam12, Myot, Defb37, Gml,* and *Ugt2b5* in the colon and *Gpr27, Gm7457, Usp2, Stfa3, Gm37800, Dync2li1, Gm32486, Gm7206, Gm33326,* and *Epha2* in blood have not been identified previously in this model. The top 10 up- and downregulated (padj ≤ 0.05, avg. exp > 100) genes in the colon and blood are listed in Supplementary Table S1.

To investigate the roles of the SDE PCGs in the UC colon and blood, we performed a functional enrichment analysis (Supplementary File S4). The top-ranked processes and pathways for the mouse colon include the immune and inflammatory system, response to inflammation, and organ/tissue remodeling (Supplementary Figure S3A). In particular, the KEGG disease pathway for IBD is enriched, with almost half of the pathway genes being SDE in our data. Although the number of SDE genes in blood is lower than in the colon, the top-ranked processes and pathways enriched for the SDE genes in blood overlap highly with the colon (Supplementary Figure S3B) and are mainly related to the immune system, inflammatory system, and connective tissue.

For the SDE lncRNAs, we performed functional enrichment of their genomic neighbors expressed in our samples (Supplementary Methods). We retrieved 888 PCGs in the colon and 159 PCGs in the blood (Supplementary File S5). Cancer, organismal injury and abnormalities, gastrointestinal disease, and immunological disease were overrepresented for both tissues, with more substantial significance in the colon than in blood (Supplementary File S5).

In the colon, for 59 out of 73 SDE miRNAs, we identified 2,330 target candidates expressed in our samples, and for blood, for eight out of 10 SDE miRNAs, we identified 1,641 targets expressed in our samples (Supplementary Figures S4A, B). In addition, functional enrichment of the targets in the colon and blood showed an overrepresentation of immune-related processes, several infectious and immunological diseases, and JAK-STAT, thyroid hormone, and MAPK signaling pathways (Supplementary File S6).

### Overlap of SDE genes in the colon and blood of the UC mouse

**FIGURE 2:**
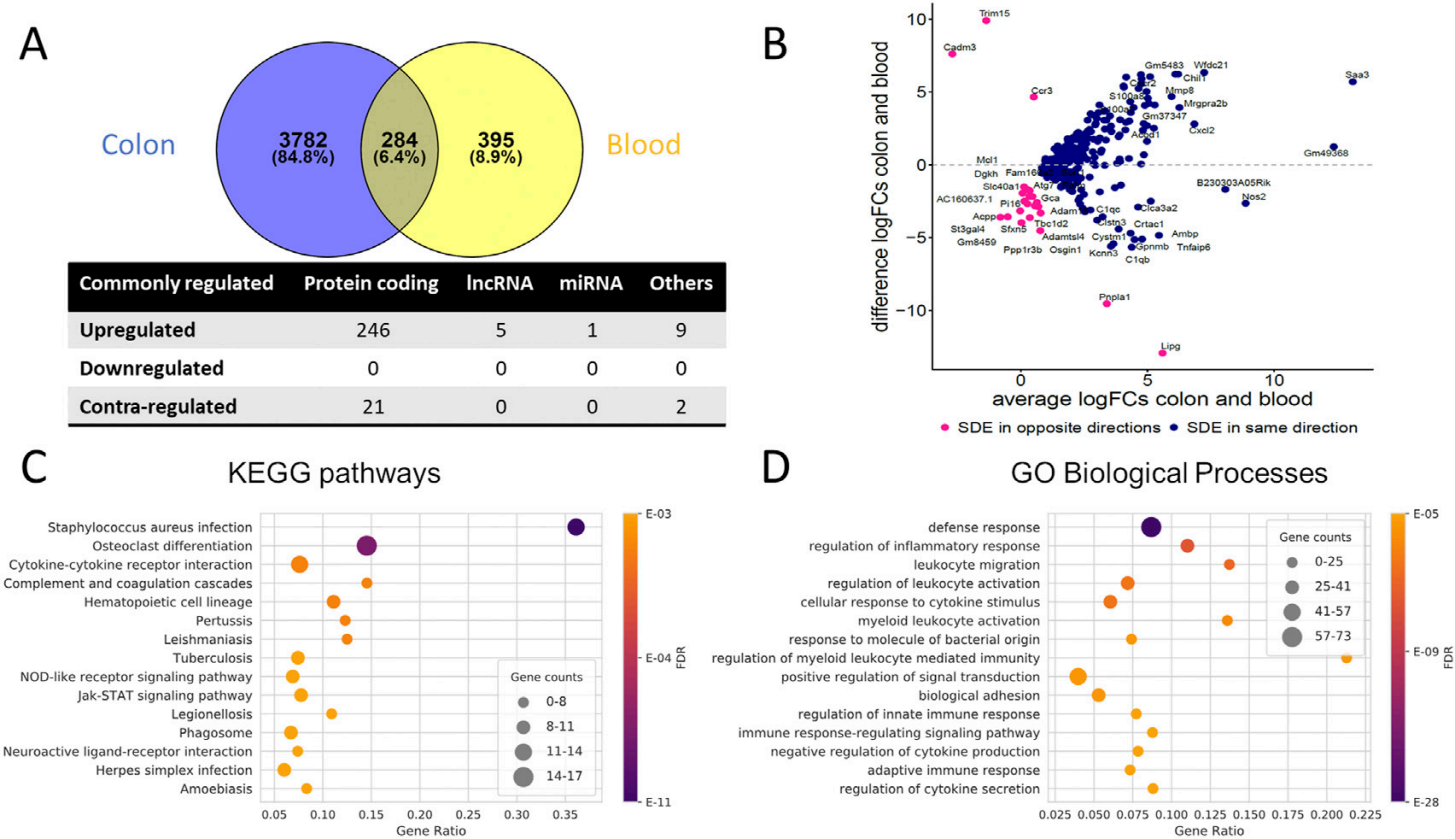
Commonly dysregulated SDE genes in UC mouse colon and blood. **(A)** Venn diagram of the SDE genes overlap between colon and blood; Other RNAs include pseudogenes, TEC, snoRNA, miscRNA, etc. Contra-regulated genes are commonly SDE genes in both tissues, which are regulated in opposite directions **(B)** MA Plot showing the relationships between log_2_FC in colon and blood for each of the common SDE genes; y-axis indicates whether the gene has a higher FC in colon or blood, x-axis reflects the cumulative FCs magnitude in colon and blood. Genes labeled as an outlier in each tissue were removed from the plot. **(C)** Top 15 enriched KEGG pathways and **(D)** Top 15 enriched GO biological processes for the common SDE genes between mouse blood and colon. Gene ratio corresponds to the ratio between the number of common SDE and all expressed genes in the pathway. The dot size indicates the number of SDE genes (gene counts), while the color represents the enrichment significance as given by the FDR adjusted *p*-values.

To identify genes commonly differentially regulated in the colon and blood of UC mice with importance in the disease phenotype, 284 genes were retrieved (Supplementary File S7). Venn diagrams and an MA plot illustrating these genes' relationship in the colon and blood are shown in Figures 2A, B. From SDE common PCGs, *Prok2* (chemoattractant, labeled as an outlier), *Saa3* (acute phase apolipoproteins), and *Gm49368* (predicted gene) were the three most upregulated genes in the colon (based on logFC), while in blood, *Gm49368* was the highest followed by *Prok2* and *Saa3*. All five common lncRNAs showed significant upregulation, with *Gm11714* most upregulated in the blood and *Mirt2* most upregulated in the colon (and second-most upregulated in the blood). For the miRNAs, miR-223-3p is the only common SDE (padj ≤ 0.05), showing upregulation in both colon and blood with higher expression in blood than in the colon, possibly indicating an immune-cell enriched miRNA. However, with a less stringent threshold of padj < 0.1, we identify three other common miRNAs downregulated in both colon and blood: miR-194-5p, miR-196b-5p, and miR-215-5p. All three are highly abundant in the colon and less abundant in blood and have previously been shown to be colon tissue-specific in humans, according to the miRNA TissueAtlas database (Ludwig et al., 2016). miR-223-3p showed to be more abundant in blood compared to the others (Ludwig et al., 2016).

Collectively, the common SDE genes define distinct inflammatory, immune system, and connective tissue-related gene expression signatures. We observed the enrichment of genes in processes related to the immune system, specifically the regulation of inflammatory response and leukocyte activation and cytokine-cytokine receptor interactions (Figures 2C, D, Supplementary Figure S3C). In addition, several KEGG pathways are related to infectious diseases (bacterial, viral, and parasitic) that alter the immune response of cells. Analysis using IPA confirms these findings (Supplementary File S7).

### Comparison between SDE genes in UC mouse colon and blood with human UC

#### Colon and blood transcriptomics signature of human UC

To obtain reliable sets of SDE genes in human UC and compare them with the mouse in both colon and blood, we identified and combined SDE genes from several public human datasets. For the colon, 2 RNA-Seq (Haberman et al., 2019) and 2 microarray studies (Mirza et al., 2015; Vanhove et al., 2015) and for blood, 2 RNA-Seq studies (Mo et al., 2018; Ostrowski et al., 2019) were used (Table 2, Supplementary File S8). Human miRNA data for the colon was obtained by combining one RNA-Seq and one microarray dataset. Unfortunately, we have not been able to identify any high-quality miRNA dataset corresponding to the whole blood of UC patients.

**TABLE 2:** Public human UC datasets used in our study.

	Accession	Description	Platform	Method	Number of samples
Colon Total RNA-Seq					
	GSE109142	Pediatric cohort, treatment-naive	RNA-Seq	Illumina TruSeq mRNA-Seq, 75 paired-End	206 UC, 20 CO
	GSE117993	Pediatric cohort, treatment-naive	RNA-Seq	Illumina TruSeq mRNA-Seq, 75 Single-End	43 UC, 55 CO
	GSE59071	Adult cohort	Microarray	Affymetrix GeneChip Human Gene 1.0 ST array	74 UC, 11 CO (colon)
	GSE67106	Adult cohort	Microarray	Agilent Custom 8 × 60K format lncRNA expression microarray	15 UC, 9 CO (colon, rectum)
Colon Small RNA-Seq					
	GSE89667	Adult cohort	Small RNA-Seq	Illumina HiSeq 2500	10 UC, 18 CO (diverticular disease)
	GSE48957	Adult cohort	Microarray	Affymetrix Multispecies miRNA-2 Array	10 UC, 10 CO
Blood Total RNA-Seq					
	GSE112057	Pediatric cohort	RNA-Seq	Illumina HiSeq 2000, 100 paired-End	15 UC, 12 CO
	PRJEB28822-C	Pediatric cohort	RNA-Seq	Ion AmpliSeq Transcriptome	34 UC, 35 CO
	PRJEB28822-A	Adult cohort	RNA-Seq	Ion AmpliSeq Transcriptome	37 UC, 32 CO

The combined sets of human SDE PCGs and lncRNAs contained ∼11,000 genes in the colon and ∼2,000 genes in the blood (padj ≤ 0.05 in at least two datasets and consistently differentially regulated across datasets). We observed approximately equal proportions of up- and downregulated PCGs in the colon and a predominant (∼75%) upregulation in blood. However, in both colon and blood, lncRNAs were mainly (∼60%) downregulated. Furthermore, ∼2,500 genes in the colon and ∼130 genes in the blood were inconsistently differentially regulated between the datasets and thus not included in the combined SDE gene sets. By combining the two public small RNA-Seq datasets in the colon (Van der Goten et al., 2014; Lin et al., 2016), we obtained 207 SDE miRNAs with padj ≤ 0.05 in at least one dataset, and 37 were SDE in both datasets. No miRNA was found to be inconsistently differentially regulated between the two datasets (Table 3).

**TABLE 3:** Numbers of SDE genes in human colon and blood combined datasets. (For all padj ≤ 0.05 in min 2 datasets).

	Colon	Blood
PCGs	▲4658 ▼4909 ✗1531	▲1382 ▼511 ✗177
lncRNAs	▲454 ▼673 ✗812	▲12 ▼16 ✗6
miRNAs	▲14 ▼19	NA
*Others	▲450 ▼181 ✗204	▲16 ▼16 ✗6

*e.g. Pseudogenes, TEC, snoRNA, miscRNA, etc. Upregulation ▲, Downregulation ▼, Inconsistent ✗.

#### Comparison between SDE genes of human and mouse UC

Using one-to-one and one-to-many orthology assignments, we compared the combined sets of SDE genes in humans with the sets of SDE genes in mouse colon and blood (Table 4). For the colon, almost 75% of the SDE mouse genes with human orthologs are also found in the human SDE set. For blood, only 30% of the SDE mouse genes with human orthologs are found in the human blood SDE gene set. The enrichment analysis for these common SDE PCGs in the UC colon and blood showed mainly immune, inflammatory, and connective tissue processes (Supplementary Files S9, 10). Seven lncRNAs showed significant up- (*H19, DNM3OS, 5430425K12RIK, 3110039I08RIK,* and *FENDRR*) and down-regulation (*HOXA11OS* and *HOTTIP*) in the colon of both mice and humans, while no common lncRNAs were detected in the blood. For five out of seven common colon SDE lncRNAs, 33 neighboring genes (100 kb upstream and downstream) were retrieved, and they were enriched for terms including connective tissue disorders, organismal injury, and abnormalities. Of the common miRNAs, twelve were downregulated, and nine were upregulated in the same direction in mouse and human colon. Functional enrichment analysis for the 1,468 targets (detected in our samples) of 17 out of 21 SDE miRNAs in the colon showed enrichment for terms including cancer, organismal injury and abnormalities, gastrointestinal disease, and several immune-related processes (Supplementary File S9).

**TABLE 4:** Numbers of overlapping genes between UC mouse and UC human colon and blood (For all padj ≤ 0.05). ⇈: Upregulation in both mouse and human, ⇊: Downregulation in both mouse and human, ⇅: Opposite directions between mouse and human

	Colon			Blood		
Human SDE genes with mouse orthologs	8446 (+ 116 miRNAs)			1723		
Mouse SDE genes with human orthologs	3066 (+ 70 miRNAs)			512		
Common SDE gens in Human and Mouse	1937 (⇈1082, ⇊360, ⇅495)			159 (⇈154, ⇊3, ⇅2)		
	⇈	⇊	⇅	⇈	⇊	⇅
PCGs	1061	345	489	154	3	2
lncRNAs	5	2	1	0	0	0
miRNAs	9	12	4	NA	NA	NA
*Others	7	1	1	0	0	0

*(e.g. Pseudogenes, TEC, snoRNA, miscRNA, etc).

Though ∼25% of common SDE genes in the colon are contra-regulated, 356 genes with upregulation in mice and downregulation in humans showed to be mainly involved in lipid metabolism, ion transport, regulation of localization, and trans-synaptic signaling. This could reflect species-specific differences in biological processes’ gene regulation. No enrichment was found for the 133 genes, downregulated in mice and upregulated in humans. *Dio3os* was the only lncRNA in the colon with upregulation in UC mice and downregulation in humans. miR-10a-5p, miR-10b-3p, and miR-10b-5p were colon miRNAs upregulated in mice and downregulated in humans and highly expressed in both. In contrast, miR-130-3p was downregulated in mice and upregulated in humans, though lower expressed in both organisms compared to the other three miRNAs (Supplementary File S9).

**FIGURE 3:**
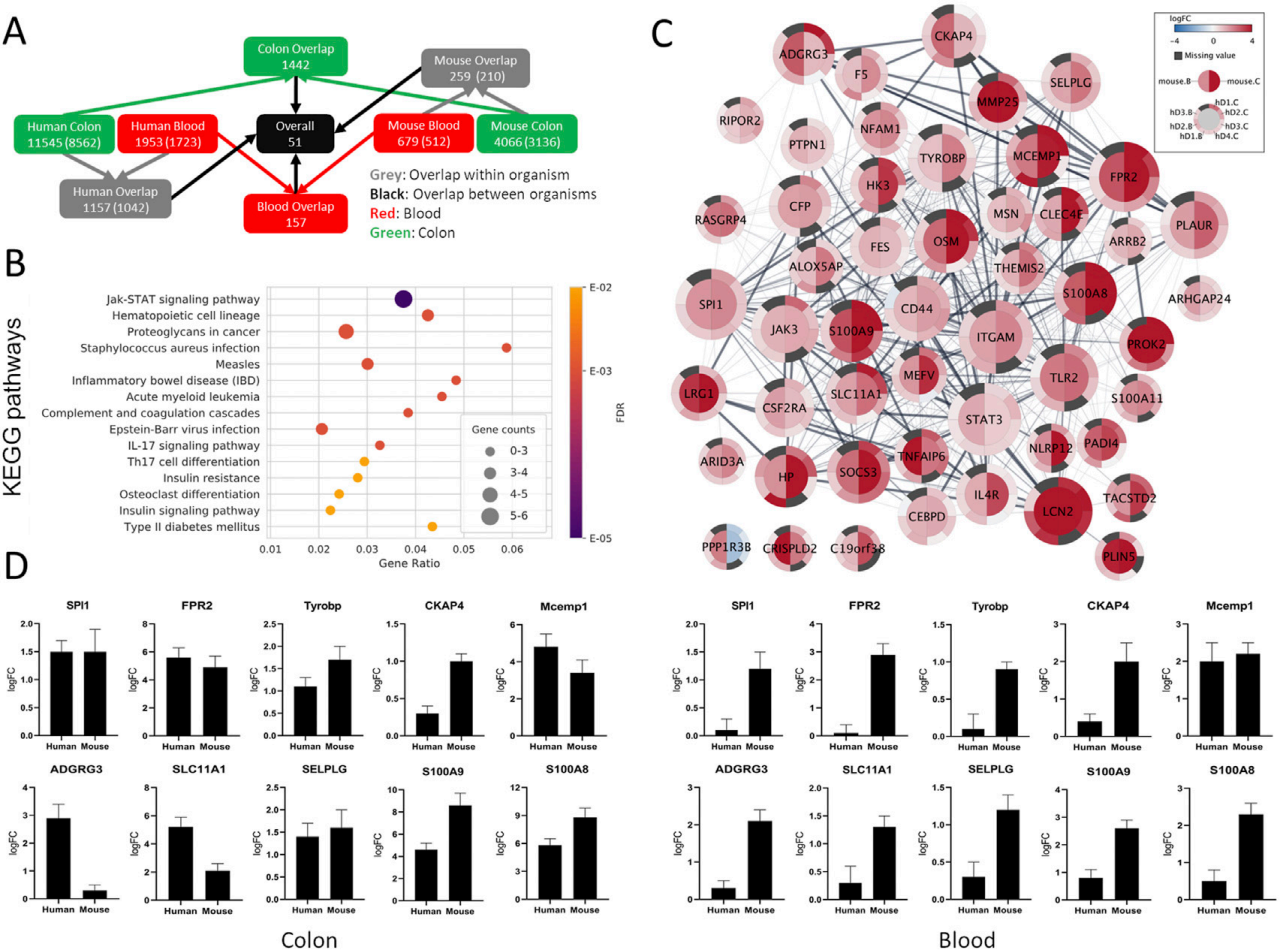
Comparison between SDE genes of human and mouse UC. **(A)** Gene overlap diagram between human combined sets and mouse colon and blood SDE genes with padj ≤ 0.05. The total number of genes in the sets and orthologous genes (in parenthesis) is shown. **(B)** Top 15 enriched KEGG pathways for the common SDE genes between human, mouse colon, and blood. Gene ratio corresponds to the ratio between the number of common SDE genes in mouse-human and all human genes in the pathway. The dot size indicates the SDE gene number (gene counts), while the color represents the enrichment significance given by the FDR adjusted *p*-values. **(C)** Differential gene expression visualization on the STRING network of genes shared between mouse (inner node circle) and human (outer node circle) in the blood (left node half) and colon (right node half). log2FC values are shown with a blue-white-red gradient, and dark grey color indicates missing values. The size of the nodes corresponds to their network importance as measured by a combination of degree, closeness, and betweenness centrality. **(D)** To validate the expression level of a few selected genes common in the colon and blood of human and mouse UC, qRT-PCR was performed. S100A8 and S100A9 were used as positive inflammatory controls.

Overall, 51 genes were identified as commonly SDE between the colon and blood of humans and mice compared with the healthy controls (Figure 3A). These genes were all upregulated except for *PP1R3B,* which is upregulated in blood and downregulated in the colon. From these genes, only *SLC11A1* and *STAT3* were IBD risk loci associated. Collectively, these strictly filtered genes showed to be mainly involved in inflammatory, immunological, and connective tissue-related processes (Figure 3B, Supplementary File S11). Neither of five lncRNAs and one miRNA, which were commonly SDE between the colon and blood of mice, were present in the human datasets.

From the 51 genes common between humans and mice in both colon and blood, 19 were selected for qPCR validation in a separate human UC cohort and the UC mice. Selected genes were either novel in a UC phenotype setting or not previously reported as therapeutic targets or biomarkers. IPA and Pharos were used to assess the therapeutic and biomarker applications of the 51 genes (Supplementary File S11). Furthermore, the selected genes were among the top central in the network of protein-protein associations retrieved from STRING v11 (Figure 3C, see ranking scores in Supplementary File S11) as measured by a combination of three complementary centrality measures (degree, closeness, and betweenness). While degree accounts for the direct interaction partners of each node, closeness and betweenness measure how central and thus important a node is with respect to all other nodes in the network (Ranking explained in Supplementary Methods). The expression pattern of all tested genes by qPCR in the colon and blood in both mouse and human validation cohorts nicely matched the RNA-seq analysis (Figure 3D and Supplementary Figure S5). The UC phenotype functional conservation and the importance for direct protein interactions with well-known immune-related and inflammatory genes suggest that these genes have high therapeutic-target and diagnostic application potential.

## Discussion

Over the last few years, several transcriptional profiling studies have provided evidence regarding the differential regulation of PCGs, lncRNAs, and miRNAs in UC (Mirza et al., 2015; Taman et al., 2018; Yarani et al., 2018; Haberman et al., 2019). Given the complex nature of UC, an *in vivo* model approach to studying its etiology is of paramount importance. The DSS-UC mouse has been extensively used as a surrogate to human UC. However, its transcriptional landscape and its (dis-) similarity to human UC have not yet been studied and comprehensively characterized.

Here, we performed colon and blood transcriptional profiling of UC mice for coding and non-coding RNAs and investigated the affected biological pathways. Our analyses showed that most differentially regulated genes are upregulated and involved in immunological and inflammatory responses and connective tissue, organ abnormality, and injuries. This points to the pathological nature of UC and the importance of inflammatory processes in destroying epithelium and aggravation of the disease. Moreover, alterations and mainly upregulation of genes that suppress inflammation were detected in the colon and blood. Genes, which demonstrated significant downregulation, were mainly genes with basic metabolic functions involved in cellular growth and proliferation, lipid, vitamin, amino acid, and mineral metabolism. Most of the genes common between the colon and blood of UC mice are regulated in the same direction (upregulated) and are related to immune or inflammatory processes. Among these genes, *S100a8, S100a9, Il1b, Lcn2, C3, Cxcl2, Nos2, Slpi, Socs3, Ifitm6, Csf2rb, Tlr2,* and *Cd44* have previously been detected in UC mouse models in other studies (te Velde et al., 2007; Hansen et al., 2009; Fang et al., 2011; Holgersen et al., 2015; Rankin et al., 2018). Several other SDE genes in the colon and blood from our study, including *Lipg, Gm5483, B230303A05Rik, Ambp,* and *Trem3,* have not been shown previously in this model. The largest overlap of 1,566 SDE genes was with a recent study by Czarnewski et al. (2019) where deep RNA sequencing was performed to stratify different subtypes of human UC using the common genes in human and mouse UC. Although they used a very similar approach, their study did not consider non-coding RNAs nor blood transcriptomes.

Here we investigated the transcriptional landscape of both lncRNAs and miRNAs in the colon and blood of the DSS-UC mice model and identified widespread differential regulation of non-coding RNAs. Numerous SDE lncRNAs and miRNAs were identified in both tissues, many of which have not been reported previously. Several SDE lncRNAs in the colon have been widely studied previously, including *H19, Meg3, Hottip, Hoxa11os,* and *Mirt2*. The latter was also SDE in blood and is a negative regulator of inflammation (Du et al., 2017). *H19* upregulation is believed to decrease the expression of the vitamin D receptor significantly and thus have a destructive effect on the intestinal epithelial barrier function by increasing permeability and decreasing the expression of ZO-1 and occludin tight junction proteins (Chen et al., 2016; Yarani et al., 2018). Not much is known regarding the other lncRNAs in UC settings, but recently *Meg3* was shown to inhibit the inflammatory response in ankylosing spondylitis characterized by chronic inflammation (Li et al., 2020). *Hottip* silencing in rheumatoid arthritis led to reduced inflammation (Hu et al., 2020). In UC mouse blood, lncRNAs *Neat1* and *Panct2* also showed to be SDE in disease. *Neat1* is shown to be proinflammatory, and its inhibition suppresses the inflammatory response in IBD (Liu et al., 2018), whereas little is known about *Panct2*. Moreover, numerous SDE lncRNAs were not reported before in UC mouse, including the top three upregulated *9130221F21Rik*, *E230034D01Rik*, *1200007C13Rik* in the colon, and *Gm7457*, *Gm37800*, *Gm32486* in blood, and many more up- and downregulated new lncRNAs were identified in our study.

Among the SDE miRNAs, several IBD-related miRNAs, including miR-30, miR-223, miR-21, and miR-142, were upregulated, and miR-192 was downregulated in the colon, while miR-223, miR-16, and miR-126 were upregulated in blood. miR-223-3p is the only miR upregulated in both colon and blood of the UC mice. It is upregulated in neutrophils and monocytes and acts as a controller of NLRP3 inflammasome activity, regulating the intestine inflammatory process by affecting IL-1β production (Neudecker et al., 2017). It has also been shown that miR-223 mediates the cross-talk between the intestinal barrier and the IL-23 pathway by targeting CLDN8, a claudin protein that constitutes the backbone of the intestinal barrier (Wang et al., 2016a; Yarani et al., 2022). miR-223 has also been used as a biomarker in IBD (Wang et al., 2016b; Yarani et al., 2022). Thus, the evidence suggests its proinflammatory role and highlights its potential as an RNA biomarker that seems to be conserved between different species. It is noteworthy that in the current study, many new SDE miRNAs are identified which are not shown in the UC mouse model previously, including the top three upregulated miR-615-3p, miR-212-3p, miR-224-5p in the colon, and miR-149-5p in blood, and many more, which need future detailed functional investigations.

By comparing our UC mouse to human UC data, we identified similar widespread directional differential regulation of genes. The overlap of a substantial number of differentially regulated genes in mouse and human UC suggests common fundamental mechanisms responsible for disease onset and progression. To date, few colon studies have compared the transcriptional changes in UC mouse models and human UC (Holgersen et al., 2015; Rankin et al., 2018; Czarnewski et al., 2019). Holgersen et al. (2015) identified 92 SDE genes in both human CD and UC, which were used to compare their three mouse models. Since colon samples were pooled in the DSS model, no statistical significance of differences could be calculated between disease and healthy mice. However, 59 genes showed to be shared between our lists. In their study, non-coding RNAs and blood transcriptomes were not studied. Rankin et al. (2018) however, identified 12 PCGs and six lncRNAs associated with DSS mouse colitis and human UC. No small RNA and blood profiling were performed in their study. In the Czarnewski et al. (2019) study, 650 SDE genes common between human and mouse UC colon were identified, but no non-coding RNA and blood profiling was performed. Apart from these studies, to the best of our knowledge, there are no human-mouse UC comparative studies considering both colon and blood and coding and non-coding RNAs to the scale of our research.

Although we recognize that separate analysis of common genes in the colon and blood may provide additional information, we focus on the genes that were common between both tissues in the mouse and human, thus, may represent the universal markers of UC. Ascribed to the pathophysiology of the UC and the fact that for diagnosis purposes, it is difficult to access the colon in comparison to blood, identification of biomarker candidates showing a similar differential expression in the colon and blood helps us diagnose UC faster and without the need for invasive assessments. Moreover, conserved same-direction differentially expressed genes in two distinct species might serve as a more reliable disease-specific biomarker compared with genes that are not expressed in one species or expressed differently in different species under the same pathogenic condition.

Overall, 51 common PCGs in mouse-human colon and blood with strong inflammatory and immunological profiles showed to be consistently differentially regulated. Several of these genes, including *ITGAM, STAT3, LCN2, TLR2, PLAUR, JAK3, CD44, OSM, SOCS3, S100A8, S100A9, HP, CFP, IL4R, FES,* and *CSF2RA*, have previously been associated with IBD. Interestingly, several less known/studied genes in IBD, including *SPI1, FPR2, TYROBP, CKAP4, MCEMP1, ADGRG3, SLC11A1,* and *SELPLG,* were identified among the top candidates. *SPI1* encodes an ETS-domain transcription factor PU.1 that activates gene expression exclusively in hematopoietic cells, including myeloid and lymphoid cells, e.g., such as lymphocyte B cell development, and is involved in disease-like Inflammatory diarrhea, primary mediastinal B cell Lymphoma and pediatric T cell acute lymphoblastic leukemia (Seki et al., 2017; Rothenberg et al., 2019). *FPR2* works as a chemoattractant receptor involved in antibacterial host defense and inflammation by sensing bacteria (Alessi et al., 2017), which is expressed not only by immune cells but also by epithelial, endothelial, and fibroblasts cells (He and Ye, 2017) that elicit proinflammatory responses. *FPR2* also promotes monocyte inflammatory activities, and its absence in knock-out mice results in increased bacteria load in the liver and reduced neutrophil infiltration (Carp, 1982). *TYROBP* encodes a transmembrane signaling polypeptide associated with the killer-cell inhibitory receptor family and plays a role in signal transduction and inflammation (Tomasello and Vivier, 2005). Loss of *TYROBP* has resulted in presenile dementia with bone cysts (Paloneva et al., 2000). *TYROBP* expression is increased in Alzheimer’s disease (AD) patients, and its deficiency in AD mice showed to be neuroprotective and immune-inflammatory therapeutic, which eventually slowed/arrested the progression of pathological late-onset sporadic AD (Haure-Mirande et al., 2017). *CKAP4* (also known as *CLIMP-63*) encodes a transmembrane protein and has been the focus of several investigations (reviewed in (Tuffy and Planey, 2012)) and is believed to regulate cell migration (Osugi et al., 2019). *MCEMP1* encodes a single-pass transmembrane protein involved in immune responses through mast cell differentiation (Li et al., 2005). *ADGRG3* has been shown to regulate the antimicrobial activity of granulocytes (Hsiao et al., 2018) and seems to be required for macrophages’ local inflammation development (Shi et al., 2016). *SLC11A1*, known as *NRAMP1*, is expressed exclusively in immune monocytes and phagocytes like macrophages (Canonne-Hergaux et al., 1999). Mutation in these genes may affect susceptibility to infections and autoimmune diseases (Sechi et al., 2006). *SLC11A1* product acidifies the phagosome (Lapham et al., 2004), thereby killing entrapped pathogens. *SELPLG*’s strong association with the immune system has been emphasized before (Tinoco et al., 2016). This gene codes for *PSGL-1* protein, a counter receptor for P-selectin that facilitates immune responses by promoting immune effector-cells trafficking into inflamed tissue. These genes are evolutionary conserved and have known interactions with other already well-known IBD candidate genes. Thus, these candidates potentially represent new UC diagnostic and therapeutic targets and novel avenues for more detailed disease mechanistic studies.

No long non-coding RNAs made it to the 51 final gene list. The major reason is that only a few lncRNAs are assigned orthology relationships across species in general (e.g., only 131 of all mouse lncRNAs are assigned a human ortholog based on the HCOP database). However, we have identified seven lncRNAs that were SDE in the colon in both humans and mice. Apart from H19, little is known about the other six lncRNAs in IBD, which clearly shows a need for detailed analysis. We also identified 21 miRNAs SDE in the colon in humans and mice. Among them, miR-146a-5p, miR -155-5p, miR-192-5p, miR-194-5p, miR-196b-5p, miR-200c-3p, miR-223-3p, and miR-223-5p were frequently shown to be differentially regulated in IBD previously, which makes them a promising candidate for further analysis.

Given the complexity of the whole colon and blood cell types, one could isolate different cell populations and perform single-cell sequencing to distinguish pathogenic mechanisms with higher resolution. One of the limitations of our study is that whole colon and blood gene expression profiling was performed. Albeit, the primary rationale was to preserve the natural state of the disease as much as possible. In addition, the disadvantages of isolation of different cell types are the technical fractionation procedures and the time duration from tissue collection to sample processing, potentially affecting gene expression statuses specifically for non-coding RNAs in cells.

In conclusion, high-throughput transcriptome analysis provides a unique tool for discovering new therapeutic and diagnostic targets by identifying relationships between gene expression and disease phenotype. Here, we showed that a one-to-one comparison of the transcriptome of the DSS-UC mouse model to human UC could provide novel disease pathophysiology information by identifying genes and pathways with essential contributions to UC. Our data suggest that prospective therapeutic interventions and diagnostic applications should target multiple major gene regulators involved in UC pathogenesis and propagations and combine several genes for valid biomarker applications. Moreover, targeting genes with conserved functional roles in the disease pathogenesis may offer a reliable UC treatment approach compared to targets with different functional roles in different tissues and organisms.

## Data Availability

The data underlying this article are available in the article and its online Supplementary Material. The raw total and small RNA-Seq data have been deposited in the Gene Expression Omnibus (GEO) database with accession number GSE155303.

## Supplementary material

Supplementary File S1 Primers for qPCR and Patients info.

Supplementary File S2 SDEGs in colon and blood.

Supplementary File S3 List of IBD loci genes.

Supplementary File S4 Functional enrichment for SDE PCGs in colon and blood.

Supplementary File S5 lncRNA-neighbors and functional enrichment.

Supplementary File S6 miRNA-targets and functional enrichment.

Supplementary File S7 Common SDEGs in mouse blood and colon.

Supplementary File S8 Confident sets SDEGs in Human UC.

Supplementary File S9 Colon common SDEGs in mouse and human.

Supplementary File S10 Blood common SDEGs in mouse and human.

Supplementary File S11 Final 51 SDEGs, centrality ranking and applications.
